# Beneficial Impacts of Fenoldopam on Patients With or at Risk for Acute Renal Failure and Undergoing Surgery: A Meta-Analysis of Randomized Clinical Trials

**DOI:** 10.7759/cureus.34584

**Published:** 2023-02-03

**Authors:** Sai Kiran Kondabolu, Yasitha Kakarlapudi, Haider Malik, Hamza Malik, Saima Khan, Praveen Kumar Komminni, Sujith K Palleti, Shamsha Hirani

**Affiliations:** 1 Internal Medicine, Andhra Medical College, Visakhapatnam, IND; 2 Internal Medicine, Shifa Tameer-E-Millat University Shifa College of Medicine, Islamabad, PAK; 3 Medicine, Foundation University Medical College, Rawalpindi, PAK; 4 Internal Medicine, Sir Syed College of Medical Sciences for Girls, Karachi, PAK; 5 Internal Medicine, Suraksha Hospital, Hyderabad, IND; 6 Nephrology, Loyola University Medical Center, Chicago, USA; 7 Cardiology, Baqai Hospital, Karachi, PAK

**Keywords:** nephroprotective effects, surgical patients, acute kidney injury, meta-analysis, fenoldopam

## Abstract

This meta-analysis aims to determine the beneficial impacts of fenoldopam on patients with or at high risk of acute kidney injury (AKI) and undergoing surgery. The Preferred Reporting Items for Systematic Reviews and Meta-Analyses (PRISMA) guidelines were followed while performing the present meta-analysis. Two investigators searched electronic databases including PubMed, EMBASE, and the Cochrane library, from inception until January 10, 2023, for relevant studies. The key terms used to search for relevant articles included “fenoldopam”, “acute kidney injury” and “surgery”. The primary outcome was the incidence of new AKI. Secondary outcomes included change in serum creatine from baseline (mg/dl), length of stay in ICU (days), renal replacement therapy (RRT), and all-cause mortality that included mortality before or at 30 days. A total of 10 studies involving 1484 patients were included in the present meta-analysis. The risk of AKI was lower in the fenoldopam group compared to the control group [risk ratio (RR): 0.73, 95% CI: 0.57-0.95]. The length of ICU stay was also shorter in the fenoldopam group [mean difference (MD): -0.35 days, 95% confidence interval (CI): -0.68, -0.03]. No significant differences were reported in terms of all-cause mortality, change in serum creatinine, and RRT. In conclusion, our meta-analysis of studies on the use of fenoldopam in adult patients undergoing major surgery showed that fenoldopam significantly reduces the risk of AKI and shortens ICU stays. However, there was no significant impact on all-cause mortality or RRT.

## Introduction and background

Acute kidney injury (AKI) is characterized by a sudden decline in the kidney's ability to filter waste products, leading to an increase in waste substances such as urea and creatinine in the bloodstream with or without changes in urine output [1]. AKI is a serious complication of high-risk surgery, linked to increased risk of morbidity, mortality, and cost of hospitalization [2]. A meta-analysis by Susantitaphong et al. found that one out of five adults experiences AKI during hospital care [3]. Current management strategies are largely preventative and consist of avoiding nephrotoxins, optimizing hemodynamics (including arterial pressure), and correcting volume depletion. At present, there are no therapeutic agents that have demonstrated efficacy in preventing or treating postoperative AKI [4].

Fenoldopam is a short-acting benzazepine selective dopaminergic A1 (DA1) receptor agonist with marginally more potent activity at the D1 receptor as compared to dopamine [5]. In contrast to dopamine, it has no α‐adrenergic or β‐adrenergic receptor activity [6]. Activation of D1 receptors in the renal arteries and afferent and efferent arterioles of the kidneys causes vasodilation and improves blood flow in the kidneys [7]. Additionally, D1 receptor activation located on the renal tubules leads to diuresis and natriuresis [8]. As fenoldopam selectively acts on DA1 receptors, it can cause more blood flow in the renal medulla compared to the renal cortex [9]. Fenoldopam may prevent AKI in high-risk surgical patients by increasing renal blood flow and reducing the energy demands of the tubules through the inhibition of sodium reabsorption. Additionally, in cases where AKI has already developed, fenoldopam may improve the resolution of AKI and prevent further kidney damage by improving renal microcirculation, particularly in the outer medulla, by removing blockages in the tubules and decreasing energy demands through inhibition of Na+/K+-ATPase. By increasing urine output, fenoldopam can also help prevent fluid overload, which is a major risk factor for poor outcomes and increased hospital resource utilization in AKI [10].

Given the lack of therapeutic agents that have demonstrated efficacy in preventing or treating postoperative AKI and the increasing need to find more effective ways to prevent AKI in high-risk surgical patients, we believe a meta-analysis of the literature on fenoldopam in this population could provide important insights into the potential benefits and risks of this intervention. This meta-analysis aims to determine the beneficial impacts of fenoldopam on patients with or at high risk of AKI and undergoing surgery.

## Review

Methodology

The Preferred Reporting Items for Systematic Reviews and Meta-Analyses (PRISMA) guidelines were followed while performing the present meta-analysis.

Search Strategy

Two investigators searched electronic databases including PubMed, EMBASE, and the Cochrane library, from inception until January 10, 2023, for relevant studies. The key terms used to search for relevant articles include “fenoldopam”, “acute kidney injury”, and “surgery”. The search was limited to randomized controlled trials (RCTs) published in the English language and conducted among adult patients undergoing surgery. Reference lists of all selected articles were also manually searched. Any discrepancy between the two authors was resolved by discussion.

Inclusion Criteria

We included all RCTs that assessed the impact of fenoldopam on patients with or at high risk of AKI and undergoing surgery. AKI was defined as per the definitions used by the authors of individual studies. We included studies comparing fenoldopam with a placebo, the standard of care, or no interventions. We excluded studies that were quasi-experimental, observational, cross-over, and those published in a language other than English. We also excluded studies that did not report targeted outcomes.

Study Selection

Retrieved results were exported to EndNote X9, and duplicate studies were identified and removed. Then, the abstracts and titles of all studies were initially screened, and irrelevant records were deleted. The full texts of all eligible records were retrieved and assessed for the inclusion and exclusion criteria. Studies that did not meet the inclusion criteria were removed. The screening results of two investigators were assessed for consistency. In case of any disagreement, a third investigator was consulted.

Data Extraction and Risk of Bias Assessment

Two authors extracted the relevant data from the included studies using a pre-defined data extraction created using Microsoft Excel. Data extracted included author names, publication year, number of patients in the intervention arm and control arm, the dose of fenoldopam, and patient characteristics. Two authors extracted the relevant data, and the third author cross-checked and entered it in Review Manager Version 5.4 for data analysis. The risk of bias was assessed using the Cochrane Risk of Bias Assessment tool for RCTs. The risk of bias of each included study was categorized into one of the three following classes: high risk, low risk, and moderate risk of bias. Any disagreement that occurred during the process of data extraction and quality assessment was resolved through discussion.

Outcomes

The primary outcome was the incidence of new AKI. Secondary outcomes included change in serum creatine from baseline (mg/dl), length of stay in ICU (days), renal replacement therapy (RRT), and all-cause mortality that included mortality before or at 30 days.

Statistical Analysis

We used Review Manager, version 5.4.0 (RevMan, the Cochrane Collaboration, Oxford, UK) to analyze data for each outcome. We analyzed binary outcomes using the Mantel-Haenszel model and calculated risk ratio (RR) with 95% confidence intervals (CIs). Mean differences (MD) were computed with their 95% CI for continuous outcomes. A two-sided p-value of 0.05 was considered statistically significant. The heterogeneity of each outcome among the study results was calculated using I^2^ statistics. An I^2^ value of <25% was considered low heterogeneity, while I^2^ >75 was regarded as high heterogeneity. We used a random-effect model if I^2 ^was more than 50%. Otherwise, a fixed-effect model was used. 

Results

**Figure 1:**
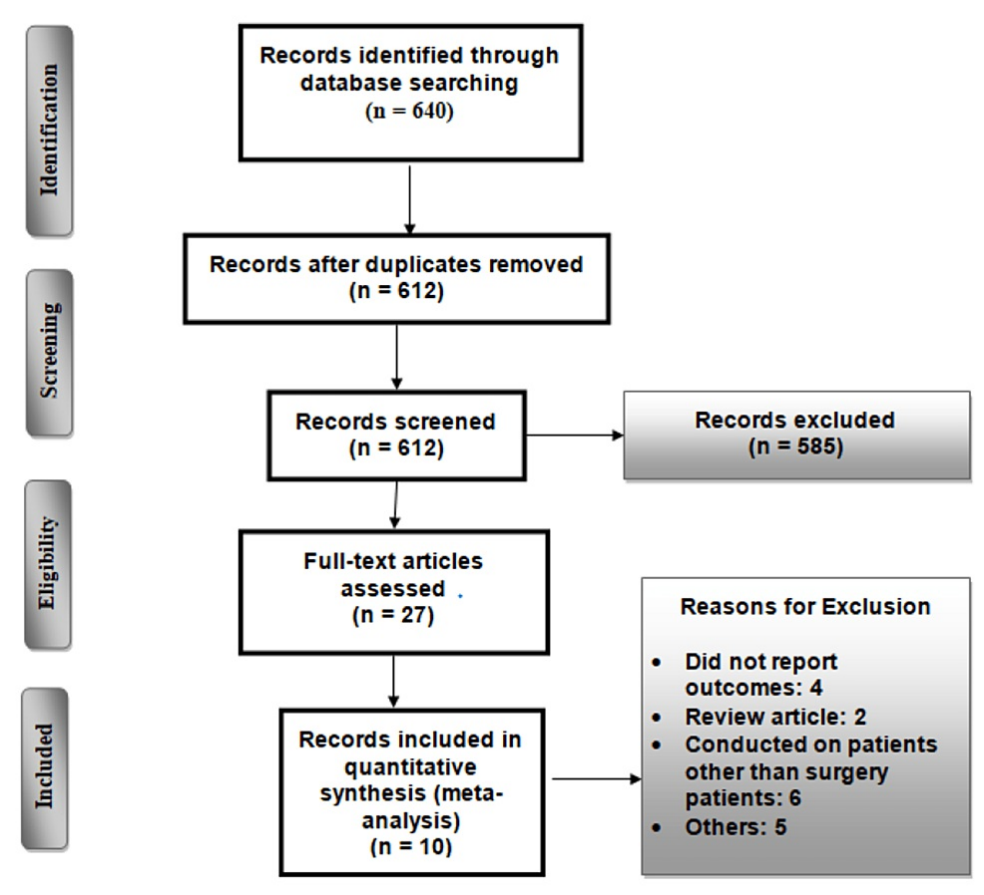
PRISMA flowchart depicting the selection of studies PRISMA: Preferred Reporting Items for Systematic Reviews and Meta-Analyses

**Figure 2:**
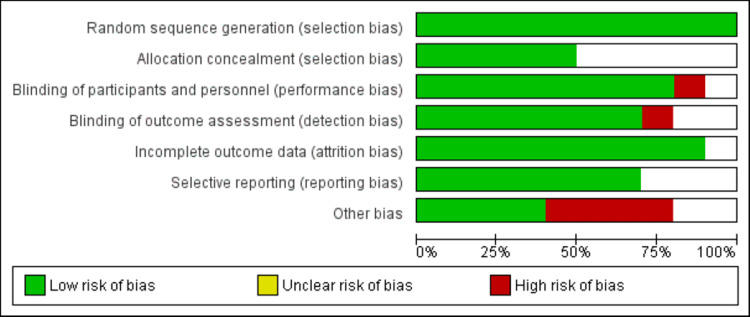
Risk of bias graph

Figure 1 shows the PRISMA flowchart of the selection of studies. A total of 640 articles were identified from electronic databases. After removing duplicates, 612 articles were assessed based on titles and abstracts. Five-hundred-eight-five articles were removed based on title and abstract screening. Full texts of 27 articles were retrieved and screened for inclusion and exclusion criteria. Ultimately, 10 studies were included in the present meta-analysis. The included studies were published from 2003 to 2014 and involved a total of 1484 patients. The main characteristics of the included studies are shown in Table 1. The majority of patients were males, and the mean age of patients in each study ranged from 48 to 75.7 years. Figure 2 shows the overall risk of bias graph.

**Table 1:** Characteristics of included studies CPB: cardiopulmonary bypass

Author name	Year	Population	Groups	Sample size	Dose and duration of fenoldopam	Mean age (years)	Males (n)
Barr et al. [11]	2008	Cardiac surgery patients with preoperative creatinine clearance of ≤40 ml/min	Fenoldopam	19	0.1 μg/kg/min for 48 hours started preoperatively	75.7	25
			Control	19			
Biancofiore et al. [12]	2004	Patients undergoing orthotopic liver transplantation	Fenoldopam	46	0.1 μg/Kg/min for 96 hours started preoperatively	48	71
			Control	46			
Bove et al. [13]	2005	Patients undergoing cardiac surgery	Fenoldopam	40	0.1 μg/Kg/min for 24 hours postoperatively	68.5	58
			Control	40			
Bove et al. [14]	2014	Patients with acute kidney injury undergoing cardiac surgery	Fenoldopam	338	0.1 μg/Kg/min for 72 hours postoperatively	70	428
			Control	329			
Caimmi et al. [15]	2003	Patients undergoing uncomplicated moderate hypothermic CPB	Fenoldopam	80	0.1-0.3 μg/kg/min for 24 hours postoperatively	69	106
			Control	80			
Cogliati et al. [16]	2007	Patients undergoing high-risk cardiac surgery	Fenoldopam	95	0.1 μg/kg/min for 24 hours postoperatively		
			Control	98			
O'Hara et al. [17]	2013	Patients undergoing partial nephrectomy	Fenoldopam	43	0.1 μg/kg/min for 24 hours postoperatively	59	62
			Control	34			
Oliver et al. [18]	2006	Patients undergoing abdominal aortic surgery	Fenoldopam	29	0.05 g/Kg/min	70.2	53
			Control	30			
Ranucci et al. [19]	2010	Patients who were scheduled for elective cardiac surgery	Fenoldopam	40	0.1 μg/Kg/min for 24 hours postoperatively	64.5	57
			Control	40			
Rocca et al. [20]	2004	Patients undergoing liver transplantation	Fenoldopam	20	0.1 μg/Kg/min	51.3	32
			Control	18			

Meta-Analysis of Outcomes

**Figure 3:**
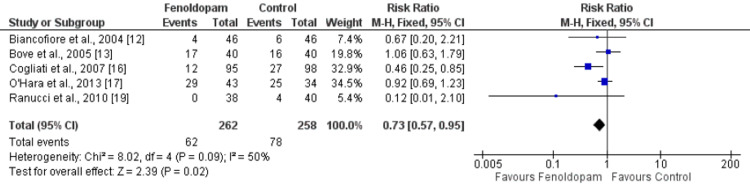
Effect of fenoldopam on the incidence of acute kidney injury* *[12-13,16-17,19]

Five studies evaluated the effect of fenoldopam on the risk of AKI compared to control groups. Our analysis found a risk ratio (RR) of 0.73 (95% CI: 0.57-0.95), indicating that the risk of AKI was 27% lower in the fenoldopam group compared to the control group. This difference was found to be statistically significant, with a p-value of 0.02. Moderate heterogeneity was found among the studies included in the meta-analysis, as indicated by an I^2^ value of 50% (Figure 3).

**Figure 4:**
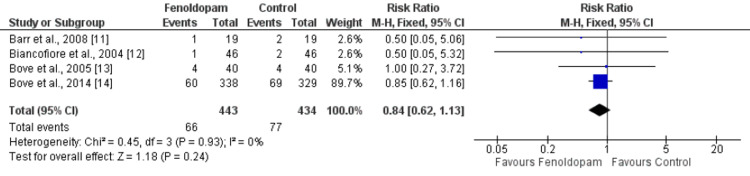
Effect of fenoldopam on renal replacement therapy* *[11-14]

Overall, four studies evaluated the effect of fenoldopam compared to controls on RRT. The pooled relative risk of RRT with fenoldopam was 0.84 (95% CI: 0.62-1.13) with a p-value of 0.24, indicating no significant difference between the two groups. The I^2^ value, a measure of heterogeneity among studies, was 0%, indicating low variability among study results (Figure 4).

**Figure 5:**
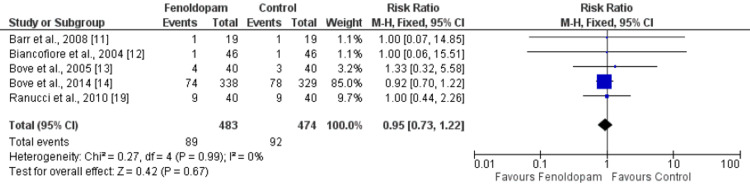
Effect of fenoldopam on all-cause mortality* *[11-14,19]

Four studies evaluated the effect of fenoldopam on all-cause mortality. The results of the meta-analysis showed that there was no significant difference in all-cause mortality between the fenoldopam and control groups. The relative risk of all-cause mortality for those treated with fenoldopam was 0.95 (95% CI: 0.73-1.22) and the p-value was 0.67. The low I^2^ value of 0% suggests that there was no heterogeneity in the results of the studies included in the analysis (Figure 5).

**Figure 6:**
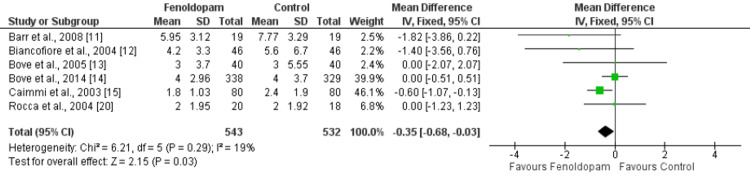
Effect of fenoldopam on ICU stay in days* *[11-15,20]

The results of our study indicate that treatment with fenoldopam is associated with a shorter length of stay in the ICU compared to controls. The mean difference in ICU stay between the fenoldopam and control groups was -0.35 days (95% CI: -0.68, -0.03), with a p-value of 0.03, as shown in Figure 6. The I^2^ statistic for this comparison was 19%, indicating low heterogeneity among the studies included in the analysis. A total of six studies were included in this comparison.

**Figure 7:**
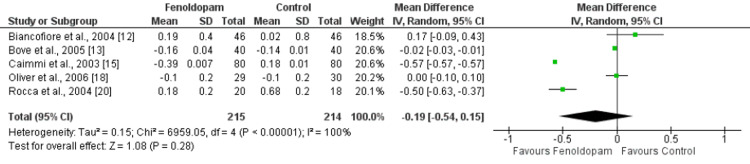
Effect of fenoldopam on change in serum creatinine from baseline* *[12-13,15,18,20]

The mean difference in change in serum creatinine levels between the fenoldopam and control groups was -0.19 (95% CI: -0.54, 0.15), with a p-value of 0.28, as shown in Figure 7. However, the I^2^ statistic for this comparison was 100%, indicating a high degree of heterogeneity among the studies included in the analysis. These results suggest that fenoldopam does not have a significant effect on serum creatinine levels.

Discussion

Our meta-analysis was conducted to determine the beneficial impacts of fenoldopam on patients with or at high risk of AKI and undergoing surgery. Our study found that treatment with fenoldopam was associated with decreased incidence of AKI. Our study also found that the length of ICU stay was shorter in patients randomized in the fenoldopam group compared to their counterparts in the control groups. Meanwhile, our study did not show any advantages of fenoldopam in reducing the incidence of all-cause mortality and renal replacement therapy.

AKI is common among hospitalized patients. A significant portion of patients who have undergone surgery, about 53%, experience AKI postoperatively. A study on patients who underwent cardiac surgery found that those who developed AKI post-surgery had a significantly higher risk of death, even up to 10 years after the surgery, even when their kidney function had returned to normal before being discharged from the hospital [21]. Similar results have also been reported for patients undergoing other major surgeries [22]. Hence, clinicians need to develop strategies to mitigate or reduce the incidence of postoperative AKI.

A previous meta-analysis conducted in 2007 found that fenoldopam significantly decreased the risk of AKI, in-hospital mortality, and RRT in intensive care patients or postoperative patients with or at high risk of AKI [23]. Another meta-analysis reported that fenoldopam was significantly associated with a significant decrease in postoperative AKI, but no significant impact of fenoldopam was observed on in-hospital mortality or RRT [4]. These meta-analyses did not consider the risk of random errors.

The meta-analysis conducted by Zangrillo et al. reported results similar to our meta-analysis. They included six studies in their meta-analysis conducted on patients undergoing cardiac surgery [24]. The findings of this meta-analysis were consistent with the results obtained by Gillies et al. [4]. Zangrillo et al. reported that the risk of AKI was lower in patients administered with fenoldopam. However, no impact of fenoldopam was observed on intra-hospital mortality and reduction of RRT [24]. The present meta-analysis is more comprehensive as it included 10 RCTs conducted among patients undergoing surgery.

The impact of fenoldopam was assessed in terms of outcomes such as the length of ICU stay in days, and changes in serum creatinine levels were also reported in our study. The reduction in serum creatinine levels was greater in patients receiving fenoldopam compared to patients in the control groups, but the difference is insignificant. Three out of five studies that assessed this outcome reported the beneficial impact of fenoldopam on serum creatinine levels [13,15,20]. Due to the small sample size and limited power, further studies need to be carried out to validate these findings.

The present meta-analysis has certain limitations. Firstly, the definition of AKI was not consistent among studies. All studies used different criteria for AKI. Secondly, none of the included studies used predefined criteria for RRT. It is vital to emphasize that the results of our study have to be considered with these limitations in mind. Moreover, in the majority of studies, the dose of fenoldopam administered was 0.1 μg/kg/min, but the duration of therapy varied among the studies.

In terms of future research, our study highlights the need for larger RCTS to further investigate the use of fenoldopam in preventing AKI in patients undergoing high-risk surgery. These studies should include a diverse patient population, clearly defined criteria for AKI, and a comparison of different dosages and durations of treatment. Additionally, it would be beneficial to investigate the use of fenoldopam in other populations, such as those with normal kidney function, and to explore the potential mechanisms of action of the drug.

## Conclusions

Our meta-analysis of studies on the use of fenoldopam in adult patients undergoing major surgery showed that fenoldopam significantly reduces the risk of AKI and shortens ICU stays. However, there was no significant impact on all-cause mortality or RRT. In terms of changes in serum creatinine from baseline, the reduction was greater in patients receiving fenoldopam, but the difference was statistically insignificant. Despite these promising findings, the limitations of the studies included, such as small sample sizes, inconsistent definitions of AKI, and variable durations of fenoldopam administration, must be acknowledged. Therefore, more well-designed RCTs are needed to further investigate the benefits of fenoldopam in preventing AKI and preserving renal function in these patients.
